# Accuracy of magnetic resonance imaging for predicting pathological complete response of breast cancer after neoadjuvant chemotherapy: association with breast cancer subtype

**DOI:** 10.1186/s40064-016-1800-x

**Published:** 2016-02-24

**Authors:** Takayo Fukuda, Rie Horii, Naoya Gomi, Yumi Miyagi, Shunji Takahashi, Yoshinori Ito, Futoshi Akiyama, Shinji Ohno, Takuji Iwase

**Affiliations:** Breast Oncology Center, Cancer Institute Hospital, Japanese Foundation for Cancer Research, Tokyo, Japan; Department of Pathology, Cancer Institute, Japanese Foundation for Cancer Research, 3-8-31 Ariake, Koto-ku, Tokyo, 135-8550 Japan; Diagnostic Imaging Center, Cancer Institute Hospital, Japanese Foundation for Cancer Research, Tokyo, Japan; Department of Medical Oncology, Cancer Institute Hospital, Japanese Foundation for Cancer Research, Tokyo, Japan

**Keywords:** Breast cancer, Neoadjuvant chemotherapy (NAC), Magnetic resonance imaging (MRI), Pathological complete response (pCR), Imaging complete response (iCR), Breast cancer subtype

## Abstract

A pathological complete response (pCR) to neoadjuvant chemotherapy (NAC) is a signature of favorable prognosis in breast cancer. The aim of this study was to assess the accuracy of magnetic resonance imaging (MRI) in predicting the pCR after NAC. 265 women with stage II or III breast cancer who underwent surgery after NAC were retrospectively investigated for MRI findings before and after the NAC. Correlation of pCR with an “imaging complete response” (iCR), defined as no detectable tumor on all serial images with dynamic contrast-enhanced T1-weighted imaging, was evaluated with respect to each tumor subtype. Of 265 cases, 44 (16.6 %) and 24 (9.1 %) were diagnosed as iCR and pCR, respectively. Nineteen of the 44 iCR cases (43.2 %) were assessed as pCR, and 216 (97.7 %) of the 221 non-iCR cases were assessed as non-pCR. The accuracy (ACC), the pCR predictive value (PPV) and the non-pCR predictive value (NPV) were 88.7, 43.2, and 97.7 %, respectively. When assessed according to each tumor subtype, the ACC, PPV and NPV were 93.2, 21.4 and 100 % for luminal subtype, 70.8, 0 and 89.5 % for luminal/HER2 subtype, 75, 57.1 and 88.8 % for HER2-enriched subtype, and 90.9, 72.7 and 97 % for triple-negative subtype, respectively. MRI is a valuable modality for predicting pCR of breast cancer after NAC treatment. However, its accuracy varies greatly in different breast cancer subtypes. Whereas MRI closely predicts pCR in the triple-negative subtype, iCR in the luminal subtype is often an over-estimation. On the other hand, residual lesions identified by MRI are reliable markers of non-pCR for the luminal subtype.

## Background

Neoadjuvant chemotherapy (NAC) is one of the recommended treatments for operable invasive breast cancer. Published clinical trials have shown that breast cancer patients with pathological complete responses (pCR) to NAC have significantly better prognosis than those without pCR (Wolmark et al. 2001; Van der Hage et al. 2001; Bear et al. 2006). Therefore, pCR is an important prognostic factor in breast cancer treated with NAC. Von Minckwitz et al. reported that the prognostic impact of pCR varied among tumor subtypes classified by their expression of hormone receptors (HRs) and human epidermal growth factor receptor 2 (ErbB2, hereinafter called HER2). Although pCR significantly correlates with favorable prognosis in triple-negative and HER2-enriched subtypes, it fails to predict the prognosis of luminal and luminal/HER2 subtypes (von Minckwitz et al. 2012).

Monitoring therapeutic responses to NAC, including early detection of progressive disease, is of pivotal importance in clinical practice. Physical examination, mammography, ultrasonography and magnetic resonance imaging (MRI) are widely used for the assessment of therapeutic effect.

MRI is a useful device to assess the size and extent of lesions in breast cancer. The size of the invasive component of the tumor estimated by MRI closely correlates with that determined pathologically. MRI is also capable of detecting small residual cancer nests after NAC (Partridge et al. 2002). Londero et al. reported that MRI could assess responses to NAC better than physical examination, mammography or ultrasonography (Londero et al. 2004). Ideally, if MRI were able to perfectly distinguish pCR from non-pCR, additional mastectomy would be avoided. The present study was conducted to determine the accuracy of MRI estimation of tumor regression in predicting pCR. We propose that assessments with respect to each tumor subtype would further enhance diagnostic accuracy.

## Methods

### Patients

A total of 2767 cases with primary breast cancer underwent surgery from January 2005 to December 2007 at the Cancer Institute Hospital of the Japanese Foundation for Cancer Research. During this period 402 patients were treated with NAC. Of these, 265 with stage II–III invasive breast cancer, no special type, where MRI had been performed before and after chemotherapy, were included in this study. In our hospital, breast cancer patients usually undergo MRI before treatment. MRI after NAC is performed preferentially in patients, where indications for breast-conserving therapy need to be assessed. We retrospectively investigated the relationship between pCR and imaging complete response (iCR) evaluated by MRI.

### Chemotherapy regimen of NAC

Standard anticancer drugs including anthracycline and/or taxane were administered to all patients as NAC. Sixty five patients received an anthracycline-based combination regimen, 22 received only taxane and the remaining 178 were treated with the anthracycline-based combination regimen followed by taxane. The former involved 4–6 cycles of CAF (cyclophosphamide 500 mg/m^2^, adriamycin 50 mg/m^2^, fluorouracil 500 mg/m^2^, q3w), AC (adriamycin 60 mg/m^2^, cyclophosphamide 600 mg/m^2^, q3w), and CEF (cyclophosphamide 500 mg/m^2^, epirubicin 100 mg/m^2^, fluorouracil 500 mg/m^2^, q3w) therapy. The taxane regimens were 12 cycles of weekly paclitaxel at a dose of 80 mg/m^2^ or 4 cycles of tri-weekly docetaxel at a dose of 75 mg/m^2^. In Japan, postoperative administration of trastuzumab was approved for health insurance coverage in 2008. In the present study, none of the patients were administered trastuzumab preoperatively.

### MRI

MRI examinations were performed using a 1.5T MRI unit (Signa HD, GE Health Care, Milwaukee, USA) and a commercially-available dedicated four-channel breast array coil. Patients underwent MRI in the prone position. Our imaging protocol included a localizing sequence followed by unilateral fast spin-echo T2-weighted coronal imaging (TR/TE, 4800/85 ms; echo train length 16, and matrix 384 × 224) with fat suppression by chemical shift-selective imaging sequences. Other parameters were as follows: field of view, 260 mm; section thickness, 3 mm and interslice gap, 0 mm. This examination was followed by combined dynamic contrast-enhanced unilateral coronal breast imaging. An enhanced T1-weighted examination 3D gradient echo sequence with fat suppression by spectral inversion recovery was performed before and after contrast material injection. The image parameters were as follows: TR/TE/FA, 3.6 ms/1.0 ms/15°; FOV, 26 × 26 cm; matrix, 320 × 240; section thickness, 3.0 mm; interslice gap, 0 mm and acquisition time, 60 s. A dynamic study in the coronal plane was performed before and 60, 120, 180 and 240 s after starting intravenous injection of 0.2 mmol/kg of gadodiamide hydrate (Omniscan^®^, Daiichi-Sankyo, Tokyo, Japan) at a rate of 3 mL/s, followed by a 20 mL saline flush at the rate of 3 mL/s.

iCR was defined as no enhanced tumor visible on any serial images of dynamic contrast-enhanced T1-weighted images. If dynamic MRI showed any amount of enhanced area, the case was diagnosed as non-iCR in this study. Non-iCR included “partial response”, “stable disease” and “progressive disease” according to the Response Evaluation Criteria in Solid Tumors (RECIST) Guidelines.

### Pathological examination of surgical specimens

Detailed evaluation of surgical specimens was carried out. Partial mastectomy specimens were cut into 5 mm thick serial sections and formalin-fixed paraffin blocks were made from the all sections. The all blocks cut into thin slices and were examined microscopically. In total mastectomy specimens, the location of the tumor bed was firstly identified from the clinical information and gross observation of the cut surfaces. Next, many tissue blocks broadly distributed around the tumor bed were sampled and microscopically examined.

pCR was defined as no invasive cancer cells in any slices of the resected breast specimen. pCR was allowed for in situ carcinoma and for positive lymph nodes.

### Subtype classification by HR and HER2 status

Tumors were classified into four subtypes based on the two HRs, the estrogen receptor (ER) and the progesterone receptor (PgR), and HER2. The status of these biomarkers was estimated using biopsy samples obtained before chemotherapy. Immunohistochemical staining for ER and PgR was performed using antibody clone 1D5 for ER and for clone PgR636 for PgR (both Dako Japan Inc., Tokyo). Positive reactions for ER and PgR were defined as nuclear staining in ≥10 % of cancer cells, and negative reactions were defined as staining in <10 %. HR-positivity was defined as positivity for ER and/or PgR. Immunohistochemical staining for HER2 was performed using the Hercep Test (Dako Japan Inc.). Expression of HER2 protein was classified into 4 strata, i.e., 0, 1+, 2+ and 3+. In the 2+ cases, HER2 genetic testing by fluorescence in situ hybridization (FISH) was performed using the PathVysion HER2-DNA Probe Kit (Abbott Molecular Inc., Des Plaines, USA). Both protein and genetic status were estimated based on the guidelines for HER2 testing in breast cancer, as recommended by the American Society of Clinical Oncology/College of American Pathologists (Wolff et al. 2013). HER2 positivity was defined as HER2 protein 3+ or HER2 gene amplification. Subtype definitions were as follows: luminal subtype, HR-positive and HER2-negative; luminal/HER2 subtype, HR-positive and HER2-positive; HER2-enriched subtype, HR-negative and HER2-positive; and triple-negative subtype, HR-negative and HER2-negative.

### Statistical analysis

Statistical analysis was performed using SPSS statistics Ver17.0 (IBM Japan Inc., Tokyo, Japan). The Fisher’s exact test was used to compare iCR and pCR. P < 0.05 was taken to indicate statistical significance in all instances.

### Ethical issues

Informed consent for the use of specimens was obtained preoperatively from all patients participating in this study, which was approved by the Institutional Review Board of the authors’ institution and was disclosed information.

## Results

### Patients’ characteristics

All patients studied were female with a mean age at the beginning of treatment of 49.9 years (range 25–78). Tumors were at Stage II (172 cases) or Stage III (93 cases) with a tumor size >2 cm (T2, 172 cases, 64.9 %; T3, 38 cases, 14.3 %; T4, 38 cases, 14.3 %) or/and positive for lymph node metastasis (199 cases, 75 %). The latter included seventeen cases of T1 with positive lymph node metastasis (6.4 %). Total mastectomy was performed on 165 patients (62.3 %) with the remaining 100 undergoing partial mastectomy (37.7 %). ER, PgR and HER2 were positive in 70.6, 46.4 and 21.1 % of cases, respectively. Data on at least one of the biomarkers were missing for 4 patients’ biopsies from other hospitals. Regarding subtype criteria combining HR and HER2, 161 cases were classified as luminal subtype (60.8 %), 24 were luminal/HER2 (9.1 %), 32 were HER2-enriched (12.1 %) and the remaining 44 were triple-negative (16.6 %).

### Comparison of iCR with pCR in the whole patient cohort

Of the total of 265 patients, 44 were diagnosed as iCR by MRI (16.6 %) and 24 as pCR (9.1 %). Of the 44 iCR patients, 19 were also diagnosed as pCR (43.2 %) and the remaining 25 as non-pCR (56.8 %). Of the 221 patients who were non-iCR, 216 were also diagnosed as non-pCR (97.7 %) but the remaining 5 were diagnosed as pCR (2.3 %). Thus, iCR was significantly correlated with pCR in the whole cohort (P < 0.001, Table 1).

**Table 1:** Comparison between iCR and pCR

	iCR	non-iCR	P value
All patients (n = 265)			
pCR	19	5	<0.001
non-pCR	25	216	
Luminal subtype (n = 161)			
pCR	3	0	0.001
non-pCR	11	147	
Luminal/HER2 subtype (n = 24)			
pCR	0	2	1.000
non-pCR	5	17	
HER2-enriched subtype (n = 32)			
pCR	8	2	0.008
non-pCR	6	16	
Triple-negative subtype (n = 44)			
pCR	8	1	<0.001
non-pCR	3	32	

*pCR* pathological complete response, *iCR* imaging complete response

Sensitivity, specificity, accuracy (ACC), pCR predictive value (PPV) and non-pCR predictive value (NPV) were estimated as 79.2, 89.6, 88.7, 43.2 and 97.7 %, respectively (Table 2).

**Table 2:** Sensitivity, specificity, ACC, PPV and NPV in each tumor subtype

	Sensitivity (%)	Specificity (%)	ACC (%)	PPV (%)	NPV (%)
All patients (n = 265)	79.2	89.6	88.7	43.2	97.7
Luminal subtype (n = 161)	100	93.0	93.2	21.4	100
Luminal/HER2 subtype (n = 24)	0	77.3	70.8	0	89.5
HER2-enriched subtype (n = 32)	80.0	72.7	75.0	57.1	88.9
Triple-negative subtype (n = 44)	88.9	91.4	90.9	72.7	97.0

*ACC* accuracy, *PPV* positive predictive value, *NPV* negative predictive value

### Comparison of iCR and pCR with respect to subtype classification

Proportions of patients with iCR in each subtype were as follows: 8.7 % (14/161) luminal, 20.8 % (5/24) luminal/HER2, 43.8 % (14/32) HER2-enriched and 25.0 % (11/44) triple-negative. In contrast, proportions of patients with pCR in each subtype were 1.9 % (3/161) for luminal, 8.3 % (2/24) luminal/HER2, 31.3 % (10/32) HER2-enriched and 20.5 % (9/44) triple-negative. Statistically significant correlations between iCR and pCR were present for luminal (P = 0.001), HER2-enriched (P = 0.008) and triple-negative (P < 0.001) subtypes, but not for the luminal/HER2 subtype (P = 1.000) (Table 1).

The sensitivity, specificity, ACC, PPV and NPV for correlations of iCR with pCR with respect to each subtype are shown in Table 2. For the luminal subtype, the NPV was 100 %, with a perfect concordance for all 147 patients with non-iCR pathologically diagnosed as non-pCR. However, only 3 of 14 iCR cases were indeed pCR, indicating a low PPV of 21.4 %. Although the sensitivity (100 %), specificity (93.0 %) and the ACC (93.2 %) were very high, these rely on the high NPV and small proportion of pCR. In contrast to luminal subtype, the PPV for the triple-negative subtype was 72.7 %, the highest score among all the subtypes, with 8 of 11 iCR cases being pCR. Sensitivity (88.9 %), specificity (91.4 %), ACC (90.9 %) and NPV (97 %) were also significantly higher for the triple-negative subtype, suggesting that MRI is a valuable modality to predict pCR for this subtype. For the HER2-enriched subtype, sensitivity (80.0 %), specificity (72.7 %), ACC (75.0 %), PPV (57.1 %) and NPV (88.9 %) were reasonably high. On the other hand, sensitivity (0 %), specificity (77.3 %), ACC (70.8 %), PPV (0 %) and NPV (89.5 %) were relatively low for the luminal/HER2 subtype.

### An example of a case diagnosed as both iCR and pCR

**Fig. 1:**
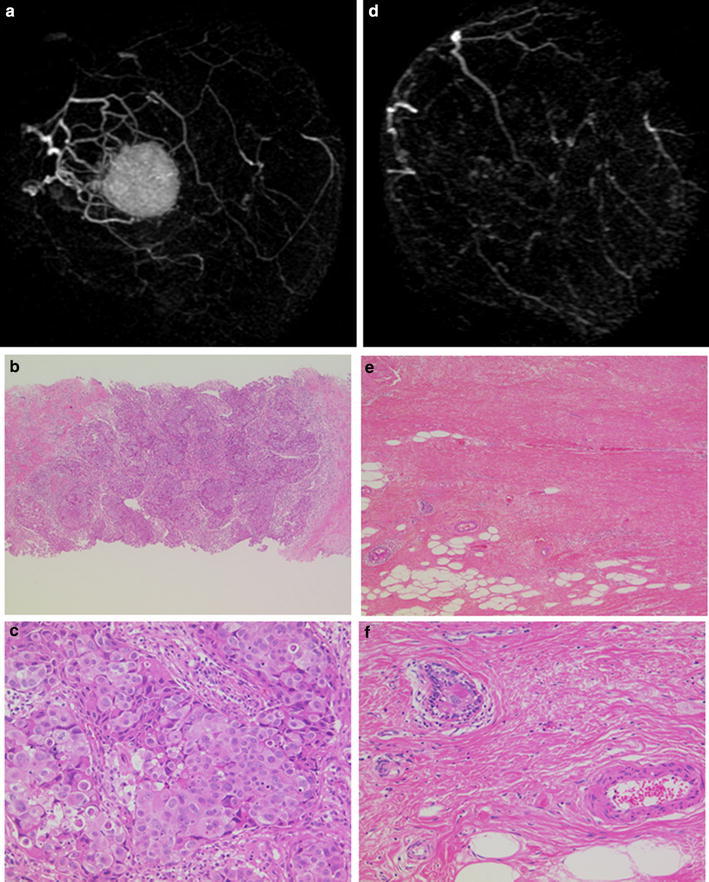
MR images and pathological findings of a case with iCR and pCR. **a**, **d** MR images of a HER2-enriched breast cancer before (**a**) and after (**d**) NAC. No enhancement was detected by MRI after NAC. **c**–**f** Histological findings of the pathological specimens before (**c**, **d**) and after (**e**, **f**) NAC. Hematoxylin and eosin staining at low (×40, **c**, **e**) and high (×100, **d**, **f**) magnification. No residual invasive cancer cells were identified. Fibrotic and granular changes are observed instead

Figure 1 shows the MR images and pathological findings of a 60-year-old patient with invasive breast cancer (T2N1M0, stage II) concordantly diagnosed as iCR and pCR after NAC. The tumor was 3.5 cm in diameter before NAC and MRI demonstrated a solid enhanced mass with a micro-lobulated margin (Fig. 1a). A core needle biopsy specimen of the tumor showed an invasive ductal carcinoma with solid growth pattern and high-grade cancer cells (Fig. 1b, c). Immunohistochemical analyses indicated that the tumor was HR-negative and HER2-positive, i.e., the HER2-enriched subtype. After NAC, no enhanced area was detectable by MRI in either early or late phases, indicating an iCR tumor response (Fig. 1d). Pathological assessment of the surgical specimen showed only fibrous granulation tissue in the tumor bed without any residual invasive cancer cells, indicating a pCR (Fig. 1e, f). A few in situ cancer cells were observed (Fig. 1f).

### Analysis of cases diagnosed as iCR but non-pCR

**Fig. 2:**
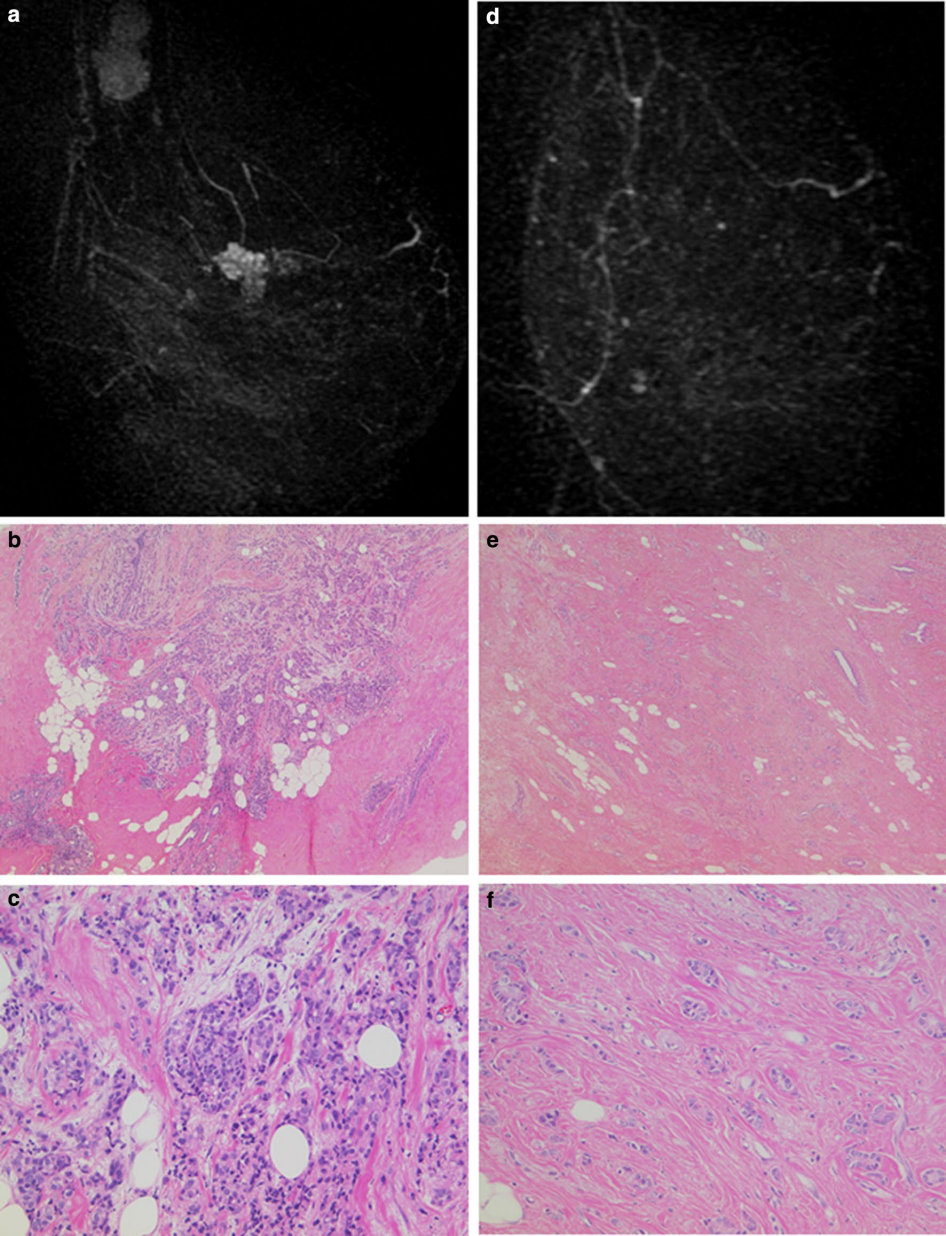
MR images and pathological findings of a case with iCR but non-pCR. **a**, **d** MR images of a luminal type breast cancer before (**a**) and after (**d**) NAC. After NAC, tumor was no longer enhanced, yielding an iCR. **c**–**f** Histological findings of the pathological specimens before (**c**, **d**) and after (**e**, **f**) NAC. Hematoxylin and eosin staining at low (×40, **c**, **e**) and high (×100, **d**, **f**) magnification. Cancer cells before NAC showed low nuclear grade. Pathology of the surgical specimen showed scattered small invasive nests of residual cancer cells in the tumor bed. Histological therapeutic effect was estimated as a “slight response”

Twenty five of 44 cases diagnosed as iCR by MRI were assessed by pathology as non-pCR (56.8 %). When assessed for subtype distribution, proportions of non-pCR in iCR cases were 78.6 % (11/14) for the luminal subtype, 100 % (5/5) for the luminal/HER2 subtype, 42.6 % (6/14) for the HER2-enriched subtype and 27.3 % (3/11) for the triple-negative subtype (Table 1). When non-pCR but iCR cases were assessed for the histological therapeutic effect according to the criteria recommended by the Japanese Breast Cancer Society (Kurosumi et al. 2008), 3 of 11 cases of luminal subtype were “slightly responsive” with the other 8 cases being “moderately responsive”, whereas only one each of the luminal/HER2, HER2-enriched and triple-negative subtypes were “slightly responsive”. On the basis of the nuclear grade of cancer cells, 6 of 11 cases of the luminal subtype were classified as low grade. The 3 “slightly responsive” patients with luminal subtype all showed a low nuclear grade. Figure 2 depicts the MR images and pathological findings of a 60-year-old patient with T1N1M0 stage II invasive breast cancer that was discordantly diagnosed as iCR but non-pCR after NAC. The tumor was 2.1 cm in diameter before NAC, and MRI demonstrated a solid enhanced polygonal mass (Fig. 2a). Here, a core needle biopsy specimen of the tumor showed an invasive ductal carcinoma with scirrhous invasion of low-grade cancer cells (Fig. 2b, c). The tumor was HR-positive and HER2-negative, the luminal subtype. After NAC, an enhanced area was no longer detected by MRI in either early or late phases; thus, the tumor response was iCR (Fig. 2d). Pathological analysis of the surgical specimen, however, revealed scattered small invasive nests of residual cancer cells in the tumor bed, indicating a non-pCR (Fig. 2e, f).

### Analysis of cases diagnosed as non-iCR but with pCR

**Fig. 3:**
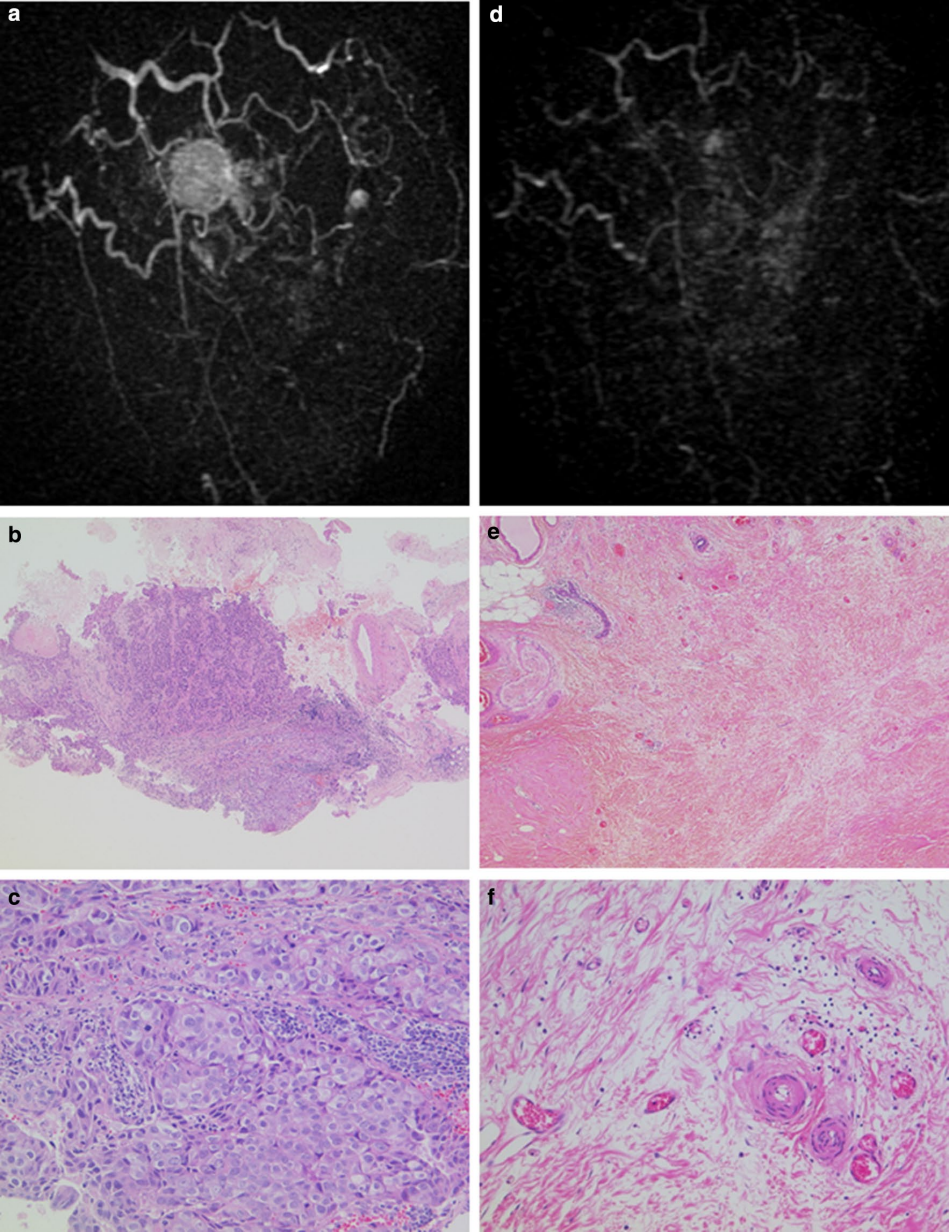
MR images and pathological findings of a case with non-iCR but pCR. **a**, **d** MR images of a HER2-enriched breast cancer before (**a**) and after (**d**) NAC. After NAC, no mass was detected but revealed scattered dendritic enhancement which predicted non-iCR. **c**–**f** Histological findings of the pathological specimens before (**c**, **d**) and after (**e**, **f**) NAC. Hematoxylin and eosin staining at low (×40, **c**, **e**) and high (×100, **d**, **f**) magnification. Pathology after surgery shows fibrous granulation tissue without residual cancer cells, regarded as a pCR

Reciprocally, we found that MRI discordantly diagnosed five cases of pCR as non-iCR. Fibrous granulation tissue that may have caused the misdiagnosis was observed in all five cases. Figure 3 shows the MR images and pathological findings of a 50-year-old patient with an invasive breast cancer (T4bN3M0, stage III) that was discordantly diagnosed as non-iCR but pCR after NAC. The tumor was 4 cm in diameter before NAC and MRI revealed a solid enhanced mass with cord-like enhancements in the lateral and the nipple side of the mass, suggesting an invasive carcinoma with ductal spread (Fig. 3a). A core needle biopsy specimen of the tumor showed an invasive ductal carcinoma with solid-tubular structure of intermediate grade cancer cells (Fig. 3b, c). Immunohistochemical analyses indicated that the tumor was HR-negative and HER2-positive, the HER2-enriched subtype. MRI after NAC showed a mass 5 mm in diameter with scattered dendritic enhancement in the later phase, signifying non-iCR (Fig. 3d). Pathology, however, showed fibrous granulation tissue in the center of the surgical specimen without residual cancer cells, indicating a pCR (Fig. 3e, f). The fibrous granulation tissue contained numerous small vessels and inflammatory cells (Fig. 3f).

## Discussion

In the present study, we investigated the accuracy of MRI for predicting pCR of breast cancer after NAC, especially focusing on its value in respect to each tumor subtype. When all cases were considered together, the iCR results were found to correlate significantly with pCR. These results are consistent with previously reported data (Straver et al. 2010; Yuan et al. 2010; McGuire et al. 2011; Hayashi et al. 2013). However, the degree of accuracy was found to differ markedly according to the tumor subtype considered. For the luminal subtype, whereas NPV was high, PPV was considerably low. This suggests that remnant lesions identified by MRI are reliable markers of non-pCR for the luminal subtype, i.e., MRI is effective for predicting non-pCR after NAC; however, iCR diagnosed by MRI in the luminal subtype is mostly an over-estimation. On the other hand, MRI accurately predicted pCR in the triple-negative subtype with a high degree of sensitivity, specificity, ACC, PPV and NPV compared to the other subtypes. These results are consistent with previous reports (Straver et al. 2010; Yuan et al. 2010; McGuire et al. 2011; Hayashi et al. 2013; Chen et al. 2014; De Los et al. 2011; Loo et al. 2011). Although a larger cohort would be required before drawing definitive conclusions, especially for the luminal and luminal/HER2 subtypes, which comprised only a small number of pCR cases, our data suggest that the overall accuracy of prediction by MRI for pCR can be enhanced by subgroup analyses. The relationship between the accuracy of MRI and subtype classification may be caused by the following points. The pCR rate of the triple-negative subtype was higher than the luminal subtype. MRI for predicting pCR is generally more accurate in tumors that have a better response. It is also known that the ER-negative tumor have higher contrast uptake on MRI after NAC than ER-positive tumor (Chen et al. 2014). In the triple-negative subtype, the high contrast uptake may result the high accuracy of MRI diagnosis.

In the present study, cases with results discordant between iCR and pCR were carefully analyzed for pathological findings. MRI diagnosed 25 cases of non-pCR as iCR. There likely to be at least two different reasons for this misdiagnosis. For HER2-enriched and triple-negative subtypes, the small volume of the residual cancer cells, often present with a scattered distribution pattern, could be responsible for their evading detection by MRI. In the cases of luminal subtype, MRI may fail to detect tumors with low nuclear grade despite a relatively large tumor size. The proportion of non-pCR in the iCR group was disturbingly high in this subtype, despite the majority of the cases having relatively large tumors and showing slight therapeutic responses. These cases all had low nuclear grade tumors. Reciprocally, MRI diagnosed five cases of pCR as non-iCR. These five shared the common findings of fibrosis and granulation occupying the tumor bed of the surgical specimen. Because the fibrous granulation tissue contained numerous small vessels and inflammatory cells, it is most likely that these NAC-responsive structures mimicked the remnant tumor in the MRI findings (Woodhams et al. 2010).

In conclusion, subgroup analysis with respect to each tumor subtype enhances the accuracy of MRI for predicting pCR after NAC treatment of breast cancer. It is an especially valuable technique for the prediction of pCR in triple-negative breast cancer. Now, we are planning a study which confirms this result with lager patients.
